# Antitumor Properties of Curcumin in Breast Cancer Based on Preclinical Studies: A Systematic Review

**DOI:** 10.3390/cancers14092165

**Published:** 2022-04-26

**Authors:** Kênia Alves Barcelos, Carolina Rodrigues Mendonça, Matias Noll, Ana Flávia Botelho, Cristiane Raquel Dias Francischini, Marco Augusto Machado Silva

**Affiliations:** 1 Postgraduate Program of Animal Science, Escola de Veterinária e Zootecnia, Universidade Federal de Goiás, Goiás 74690-900, Brazil; anafmb@ufg.br (A.F.B.); cristianeraquel@unirv.edu.br (C.R.D.F.); 2 Faculty of Medicine, Universidade de Rio Verde—UniRV, Rio de Janeiro 75901-970, Brazil; 3 Graduate Program in Health Sciences, Escola de Medicina, Universidade Federal de Goiás, Goiás 74605-050, Brazil; carol_mendonca85@hotmail.com (C.R.M.); matias.noll@ifgoiano.edu.br (M.N.); 4 Instituto Federal Goiano (IF Goiano), Goiás 74270-040, Brazil; 5 Department of Sports Science and Clinical Biomechanics, University of Southern Denmark, 5230 Odense, Denmark

**Keywords:** turmeric, anticancer, breast tumor, in vitro, in vivo, nanoparticles

## Abstract

**Simple Summary:**

Natural formulations and phytotherapies have shown promising antitumor activities. This review assesses the antitumor effects of curcumin on breast cancer. In particular, we discuss the effects of curcumin on the proliferation, viability, and apoptosis of breast cancer cell lineages and tumor volume. Studies have shown that curcumin administered at different concentrations inhibited proliferation, decreased viability, and induced apoptosis in human and animal breast cancer cells. Nanoparticle formulations of curcumin administered orally, via implant, or intraperitoneally reduced the tumor volume of human and murine mammary cells in vivo. Moreover, curcumin nanoformulations facilitate tumor growth inhibition in animal models of breast cancer. Randomized clinical trials are warranted to assess the efficacy and safety of curcumin formulations for clinical use.

**Abstract:**

Breast cancer is one of the most common neoplasms among women. Anticancer strategies using natural formulations and phytotherapies are promising antitumor treatment alternatives. This review assesses the antitumor effects of curcumin on breast cancer reported in preclinical in vitro and in vivo animal models. We used five databases to search for preclinical studies published up to May 2021. The assessments included the effects of curcumin on the proliferation, viability, and apoptosis of breast cancer cell lineages and on tumor volume. In total, 60 articles met the inclusion criteria. Curcumin administered at different concentrations and via different routes of administration inhibited proliferation, decreased viability, and induced apoptosis in human and animal breast cancer cells. Nanoparticle formulations of curcumin administered orally, via implant, and intraperitoneally reduced the tumor volume of human and murine mammary cells in vivo. Moreover, curcumin nanoformulations exert positive effects on tumor growth inhibition in animal models of breast cancer. Further randomized clinical trials are warranted to assess the efficacy and safety of curcumin formulations for clinical use.

## 1. Introduction

Breast cancer is one of the most common neoplasms in women and an important public health problem worldwide [1]. Breast cancer has surpassed lung neoplasm as the most frequently diagnosed cancer, with approximately 2.3 million new cases reported (11.7%) in 2020, according to the Global Cancer Observatory [2]. 

Conventional treatment for breast cancer includes surgical resection, radiotherapy, and chemotherapy [3]. In addition, promising alternative approaches, such as targeted therapy, immunotherapy, and hormone therapy, are currently under investigation [4,5]. These therapies vary in their mechanisms of action. The appropriate treatment regime is determined based on the type of tumor, disease stage, and clinical condition of patients [4,5].

Although chemotherapy remains the gold standard for treating several types of cancer, severe adverse reactions and tumor resistance to treatment and hormone therapy are considered negative aspects of paramount importance [3,6]. Therefore, alternative anticancer therapeutic strategies, such as the use of low-toxicity natural subproducts and extracts, are promising modalities [6,7].

Previous studies have reported that curcumin, a turmeric-derived phytochemical, exhibits beneficial biological activities, including antibacterial, antiviral, anticancer, anti-inflammatory, and antioxidant properties, and was found to exert preventive and therapeutic effects in various cancers, including breast cancer [7,8,9,10,11,12]. However, the therapeutic applicability of curcumin remains limited owing to its low water solubility and bioavailability [7,13]. Only two systematic reviews on the effects of curcumin on breast cancer have been reported to date [13,14]. Gianfredi et al. [14] investigated the bioactive effects of a curcumin-containing diet on human breast cancer cell lines [14]. Meanwhile, Ombredane et al. [13] reported the in vivo efficacy and toxicity of curcumin nanoparticles (CUR-NPs) as a treatment strategy against breast cancer. Therefore, there remain gaps in the literature regarding the effects of curcumin on tumors. In this systematic review, we collated data from preclinical in vitro and in vivo studies conducted on animal models to investigate the effects of curcumin on the proliferation, viability, and apoptosis of breast cancer cells and tumor volume, focusing on dose and administration route. We systematically reviewed the antitumor effects of curcumin on breast cancer previously reported. To our knowledge, this is the first systematic review of in vitro preclinical studies on the effect of curcumin on breast cancer cell lineages and animal models of breast cancer.

## 2. Materials and Methods

### 2.1. Protocol and Registration

The study design was based on the guidelines of the Preferred Reporting Items for Systematic Reviews and Meta-Analyses [15] and the Systematic Review Center for Laboratory Animal Experimentation (SYRCLE) [16]. A protocol was published in the International Prospective Register of Systematic Reviews: Review of animal data from experimental studies databases (CRD42021256605). We included data from preclinical in vitro and in vivo studies conducted using animal models.

### 2.2. Search Strategy and Eligibility Criteria

The PubMed, Embase, Scopus, Web of Science, and SciELO databases were used for data retrieval. The research period was limited to 23 May 2021. Google Scholar and the reference lists of primary studies were consulted to search for additional studies. The following uniterms were used: “Curcumin”; “Curcuma longa”; “Turmeric”; “Natural yellow 3”; “Turmeric yellow”; “Indian saffron”; “Kacha haldi”; “Curcumin Nanoparticles”; “Breast Cancer”; “Breast Neoplasms”; “Triple Negative”; “Breast Neoplasms”; “Breast Tumor”; “Inflammatory Breast Neoplasms”; “Carcinoma, Ductal, Breast”; “Carcinoma Lobular”; “Her-2 Positive”; “Breast Cancer”; “In Vitro”; “Mouse”; “Animal”. The search strategy adopted for each database is listed in (Table S1).

The participants, intervention, comparator, and outcomes (PICO) framework was used to determine the eligibility criteria for the systematic review of preclinical animal studies, as follows:

- Patient: laboratory animals with induced breast cancer (all species).
- Intervention(s): curcumin
- Comparator(s): control group or comparison with no treatment, treatment with other drugs, and/or traditional radiotherapy or chemotherapy regimens.
- Outcomes: antitumor activity (reduction in tumor volume and dimensions) in in vitro studies.

The inclusion criteria were as follows: (1) in vitro and animal experimental model investigations on the effects of curcumin on human and animal breast cancer cells of different lineages, (2) peer-reviewed original research articles, (3) no language restrictions, and (4) no publication year restriction. The exclusion criteria were as follows: (1) doctoral and master’s theses, (2) case studies, (3) editorials, (4) letters to editor, (5) duplicate studies found in more than one database and in silico studies, (6) epidemiological studies, (7) clinical assays and articles that requested permission from authors without response, (8) studies irrelevant to the antitumor effect of curcumin on breast cancer, (9) trials performed in non-oncological clinical conditions, (10) studies involving a sole treatment protocol based on the association between curcumin and other treatment modalities, and (11) trials involving immunodeficient animal models.

#### Definitions

Cell proliferation: increase in cell count owing to cell division [17]. Cell proliferation was strictly controlled without any alterations. In contrast, neoplastic cells exhibited massive and uncontrolled proliferation [18].

Cell viability: quantification of viable cells for estimating cytotoxicity [19] and investigating cell activity and integrity [19,20]. 

Apoptosis: programmed cell death under physiological and pathological conditions [21,22]. In cancer, disparity between cell replication and death causes malignancy [22].

### 2.3. Review Process

Two authors (K.A.B. and C.R.M.) performed a peer review of the titles and abstracts of the articles using Rayyan software. The selected articles were assessed by the authors and critically evaluated based on the known antitumor effects of curcumin on breast cancer. Next, the selected articles were assessed and the inclusion/exclusion criteria were applied. Doubts and disagreements regarding article selection were discussed with the research team. If some published studies were associated with the same project or were retrieved from the same database, the most complete study was selected [23,24].

### 2.4. Training of Reviewers

The authors participating in eligibility assessments completely understood each step of the review process, primarily the inclusion/exclusion criteria, and practiced eligibility assessments on 50 test abstracts prior to coding articles. The authors also used risk-of-bias instruments and performed quality assessments and data extraction on five articles that were not included in the review [23].

### 2.5. Evidence Synthesis

The following data were extracted: authorship, year of publication, country, cell lineage, concentration, exposure time, animal experimental model, follow-up, sample, dosing, route of administration, and main outcomes. The outcomes included antitumor activity, including cell proliferation, viability, apoptosis, and/or cessation of the cell cycle in in vitro studies and changes in tumor volume and magnitude in animal models.

The Grading of Recommendations Assessment, Development, and Evaluation (GRADE) tool, adapted for in vitro study designs [25], was used to assess quality, since methods specifically for this purpose are lacking. In vitro trials were ranked as “high,” “moderate,” and “low” in terms of quality [25] based on the analysis of each study. 

The SYRCLE RoB Toll tool was used to assess the quality of animal model studies [26]. Selection, performance, detection, attrition, reporting, and other biases were investigated.

There was substantial heterogeneity among the studies, which was detrimental to meta-analysis. Therefore, narrative synthesis was performed without statistical or sensitivity analysis, assessing publication bias using the funnel plot and Egger’s and Begg’s tests.

## 3. Results

**Figure 1:**
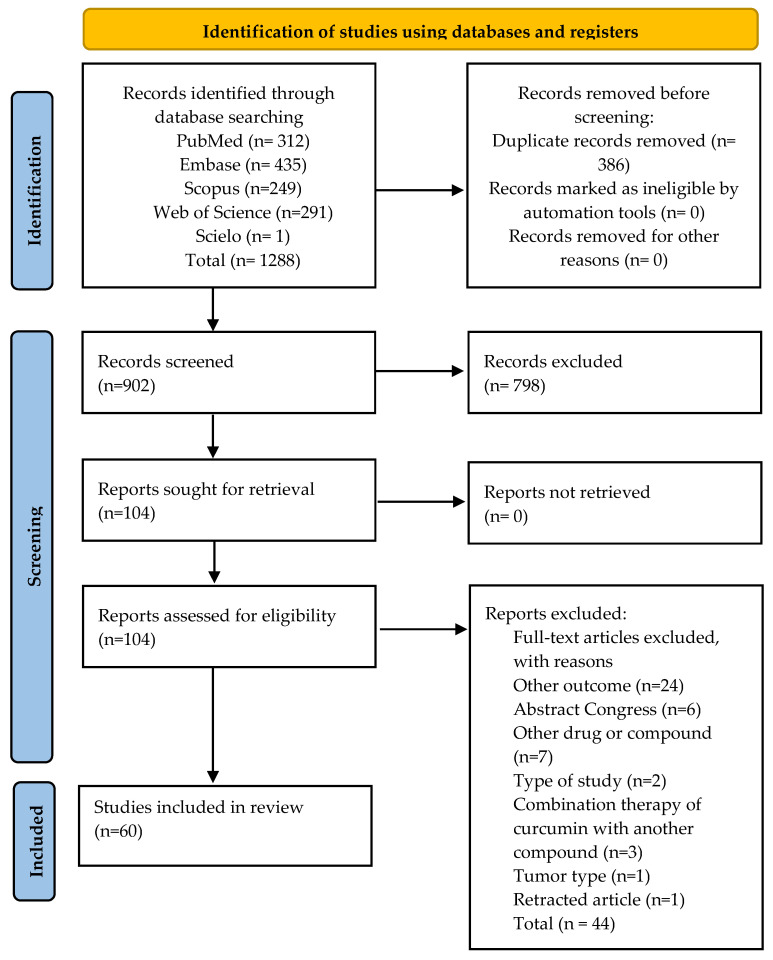
Flowchart for study selection (PRISMA Flow Diagram 2020).

The bibliographic survey yielded 1288 articles. After titles and abstracts from the records were screened, 104 potentially eligible articles were identified and selected for complete reading. Following a review of all texts, 44 articles were excluded. Details of the search strategies are provided in (Table S1). The reasons for exclusion are provided in (Table S2). The flowchart of the study is shown in Figure 1.

### 3.1. General Characteristics of the Studies

Sixty studies on the effect of curcumin on breast cancer [3,7,27,28,29,30,31,32,33,34,35,36,37,38,39,40,41,42,43,44,45,46,47,48,49,50,51,52,53,54,55,56,57,58,59,60,61,62,63,64,65,66,67,68,69,70,71,72,73,74,75,76,77,78,79,80,81,82,83,84] were included in this investigation, with 23 in vitro trials [27,28,31,33,34,35,36,37,44,45,48,51,52,54,59,64,65,70,73,74,76,80,84], 20 studies on animal models [29,30,32,40,41,46,57,60,62,66,68,69,71,72,75,77,79,82,83], and 17 studies with both in vitro and in vivo experimental designs [3,7,39,42,43,47,49,50,53,55,56,58,61,63,67,78,81]. The oldest and most recent articles were published in 1997 [64] and 2021 [39,81], respectively. The general characteristics of the selected articles are presented in Table 1 and Table 2.

### 3.2. Summary of the Results

#### 3.2.1. In Vitro Studies

Forty studies were conducted using in vitro design and assessment (Table 1) [3,7,27,28,31,33,34,35,36,37,39,42,43,44,45,47,48,49,50,51,52,53,54,55,56,58,59,61,63,64,65,67,70,73,74,76,79,80,81,84]. 

The human breast cancer cell lineages used in the studies were as follows: MCF-7 [27,31,34,36,42,44,48,49,51,56,64,65,70,76,78,79], MDA-MB-435 [45,63], T47D [35,44], MCF-7/LCC2 [48], LCC9 [48], MDA-MB-468 [50,63], and BT-474 [63]. 

Moreover, studies conducted using the triple-negative breast cancer cell line MDA-MB-231 [3,28,31,33,36,37,39,47,52,53,54,56,59,61,63,67,73,74] and human breast cancer cell lineage expressing the Her2 SK-BR-3 gene [63,80] were also assessed. In animal models, murine mammary carcinoma 4T1 [43,53,58,81] and H-2” (TUBO) [63] cell lineages were investigated. 

#### 3.2.2. In Vitro Cell Proliferation

The in vitro proliferation of breast cancer cell lineages was assessed using a quantitative image assessment technique [34], Transwell assay [42], colony formation assay [48,50], sulforhodamine B, colorimetric analysis [56], method (NR) for determining inhibition of cell growth [59], thymidine incorporation assay [3H], flow cytometry tests [64], and MTT assay, as described in most studies (Table 1).

The effect of curcumin on cell proliferation was investigated only in human cell lines. Curcumin administered at concentrations of 1, 3, 10, 20, 30, and 50 μg/mL for 24 h inhibited the proliferation of MCF-7 cells, with growth recurrence in the subsequent 72 h [27,34,42,44,56,64,76]. Optimal inhibition was achieved upon treatment with a single dose of 25 μM curcumin for 24 h [34]. A substantial reduction in growth was observed in malignant MCF-7 cell lines, with 37%, 54%, and 73% reduction upon treatment with 20, 50, and 100 μM curcumin, respectively [84].

Proliferation in MDA-MB-435 cell lineages was inhibited following treatment with 0, 10, 25, 50, and 75 μM curcumin [45,63]. The formation of colonies from MCF-7/LCC2 cells was inhibited following treatment with 30 μM curcumin [48]. The number of colonies in MDA-MB-468 cell cultures reduced over two weeks upon treatment with 5 µM curcumin [50]. Likewise, the proliferation of BT-474 cell cultures was inhibited upon treatment with 10 μg/mL curcumin [56]. In studies on triple-negative MDA-MB-231 cell lineages, cell proliferation was inhibited upon treatment with 0, 1, 25, 2,5, 5, 10, 15, 20, 30, and 50 μM curcumin for 24 and 48 h [7,28,33,59,73]. Furthermore, MSN-curcumin nanoparticles exhibited anticancer properties at 20 μg/mL [39].

**Table 1:** Characteristics of the in vitro studies included in the systematic review on curcumin and breast cancer.

Author/Year/Country	Type of Cell/Model	Intervention		Outcomes			Conflict of Interest
				Antitumor Activity			
		Concentration (Component)	Treatment Duration	Cell Proliferation In Vitro	Cell Viability	Apoptosis and/or Cell Cycle Interruption	
Abbaspour and Afshar, 2018 [27] Iran	MCF-7 Human	Curcumin at 10, 20 and 30 μg/mL	24, 48, and 72 h	MTT assay ↓ cell proliferation owing to downregulation of ODC1 and ADA gene expression.	MTT assay ↓ viability of cells in a time- and dose-dependent manner.	Not reported	None
Abuelba et al., 2015 [28] Romania	MDA-MB-231 Human	Curcumin at 15–19 μM	24, 48, and 72 h	MTT assay ↓ cell proliferation upon treatment with 15 μM curcumin.	MTT assay ↓ cell viability by up to 25% upon treatment with 15 μM curcumin.	MTT assay Pro-apoptotic effects on MDA-MB-231 cells cultured in a single layer, without photoactivation.	None
Bimonte et al., 2015 [7] Italy	MDA.MB231 Human	Curcumin at 10 and 50 μM	48 h	MTT assay Inhibition of breast cancer cell migration in 48 h. ↓ cell proliferation (*p* < 0.05).	Not reported	Flow cytometry Curcumin (10 μM) ↑ apoptosis (*p* < 0.0001).	None
Calaf et al., 2018 [31] Chile	MCF7 MDA-MB-231 Human	Curcumin at 30 µM	48 h	Not reported	Not reported	Flow cytometry Apoptosis MDA-MB-231: 14.2% MCF7: 4.6%	None
Chiu and Su, 2009 [33] China	MDA-MB-231 Human	Curcumin at 10, 20, and 30 μg/mL	48 h	MTT Assay ↓ proliferation of MDA-MB-231 cells via p21 expression.	Not reported	Flow cytometry Curcumin induced apoptosis via positive regulation of the Bax:Bcl-2 ratio.	None
Choudhuri et al., 2002 [34] India	MCF-7 Human	Curcumin at 10 and 25 μM	24 h	Quantitative image analysis Cessation of cell growth followed by significant cell death. Optimal inhibition was obtained upon treatment with 25 μM curcumin.	Not reported	Quantitative image analysis techniques Curcumin induced apoptosis.	None
Coker-Gurkan et al., 2019 [35] Turkey	T47D Human	Curcumin at 30 µM	24 and 48 h	Not reported	MTT assay ↓ cell viability by 48% and 60% upon treatment with 20 µM curcumin (*p* < 0.0024).	Double staining with Annexin-V/PI Curcumin induced apoptosis in 10.9% and 5.2% of the cell populations.	None
Coker-Gurkan et al., 2018 [36] Turkey	MCF-7 MDA-MB-231 Human	Curcumin at 30 µM	24 and 48 h	Not reported	MTT assay ↓ cell viability MCF-7 cells by 49% and of MDA-MB-453 cells by 48% upon treatment for 24 h with 20 µM curcumin	MTT assay Curcumin induced apoptotic cell death.	None
Fan et al., 2016 [37] China	MDA-MB-231 Human	Curcumin at 50 μg/mL	24 h	Not reported	MTT assay ↓ cell viability (% NR) (P:NR)	MTT assay Curcumin induced apoptosis.	None
Ghosh et al., 2021 [39] India	MDA-MB 231 Human	Curcumin at 50 μg/mL Nanostructured platform Nanoparticles, MSN-Curcumin (MSN-C), and MSN-Hyaluronic acid-Curcumin (MSN-HA-C)	48 h	MTT Assay MSN-HA-C blocked cell proliferation, in contrast to free curcumin. The treatment agent exhibited anticancer properties at 20 μg/mL.	Not reported	MTT assay Cell death MSN-HA-C: 58% MSN-C: 34% (with equivalent dose of 12 μg/mL curcumin). MDA-MB-231 cycle arrest ↓ G1-phase cells: 32.5% Control: 54.6% G2/M phase cells: 37.8% Controls: 11.4%.	None
Hashemzehi et al., 2018 [42] Iran	MCF-7 Human	Curcumin at 1 mM Nanostructured platform Nano-curcumin—phytosomalcurcumin	24 h	Transwell assay ↓ cell invasion MTT assay ↓ cell growth in a dose-dependent manner.	Not reported	Not reported	None
He et al., 2019 [43] China	4T1 Mouse	Curcumin at 50 μg/mL Nanostructured platform Polymeric micellar NPs [amphiphilic diblock copolymer—mPEG-b-PLG (Se) -TP]	48 h	Not reported	MTT assay ↓ of cell viability upon treatment with CUR-NP and Free CUR: 15%	Not reported	None
Hu et al., 2018 [44] China	T47D, MCF7 Human	Curcumin at 10 or 30 µM	72 h	MTT assay ↓ cell proliferation	Not reported	Flow cytometry Apoptosis T47D cells: 13.87% and 30.09%. MCF7 cells: 15.14% and 35.04%.	None
Hua et al., 2010 [45] China	MDA-MB-435 Human	Curcumin at 10, 25, 50, and 75 μM	12, 24, or 48 h.	MTT assay ↓ cell proliferation, inducing arrest in the G1 phase.	Not reported	Not reported	NR
Ji et al., 2020 [47] China	MDA-MB-231 Human	Curcumin at 50 μg/mL	24 h	Not reported	Not reported	Flow cytometry Apoptosis HA@CUR-NCs 80%.	None
Jiang et al., 2013 [48] China	MCF-7/LCC2 and LCC9 Human	Curcumin at 10 and 30 μM	24, 48, 72, and 96 h	Colony formation assay ↓ colony formation Complete suppression of colony formation upon treatment with 30 μM curcumin.	Not reported	Annexin-V/PI staining and flow cytometry 30 μM curcumin caused a significant increase (28.72% in MCF-7 cells, 31.36% in MCF-7/LCC2 cells, and 34.70% in MCF-7/LCC9 cells) in the percentage of late apoptotic cells.	None
Jin et al., 2017 [49] China and USA	MCF-7 Human	Curcumin at 10 µg/mL Nanostructured platform CUR-NP; GE11-CUR-NP; Free CUR	24 h	Not reported	Nanostructured platform CUR-NP, GE11-CUR-NP, and Free CUR	Flow cytometry Apoptosis CUR-NP: 14.9%; GE11-CUR-NP: 18.9%; Free CUR 11.0%.	None
Jung et al., 2018 [50] South Korea	MDA-MB-468 Human	Curcumin at 5 and 10 μM	72 and 96 h	Colony formation assay ↓ number of colonies over 2 weeks to 36.9 ± 7.7% upon treatment with 5 µM curcumin.	Unclear method ↓ significantly decreased cell viability (41.5 ± 2.8% of basal level) upon treatment with 10 µM curcumin	Not reported	None
Kim et al., 2012 [51] Coreia do Sul	MCF-7 Human	Curcumin at 1, 5, 10, 30, and 50 μM	24 h	Not reported	MTT assay Curcumin exerted no effect on the viability of MCF-7 cells	Not reported	None
Kumari et al., 2017 [52] India	MDA-MB-231 Human	Curcumin at 50 and 100 μg/mL Nanostructured platform free CUR and CUR-mPEG-PLA-Ch micelles	24 h	Not reported	MTT assay CUR: 55.26 ± 3.7% Free CUR: 66.84 ± 2.4% (*p* = 0.079)	Not reported	None
Kumari et al., 2020 [53] India	MDA-MB-231 Human 4T1 Mouse	Curcumin at 50 μg/mL Nanostructured platform CUR treatment (Free CUR group—24 μg/mL) and CUR-HSA-DOPE NPs treatment (CUR-HSA-DOPE group)	6 and 24 h	Not reported	MTT Assay MDA-MB-231 Cur-HSA-DOPE NPs 24.34 ± 6.1% and 33.99 ± 4.5% free CUR 34.87 ± 4.9% and 43.12 ± 2.4% 50 μg/mL curcumin 4T1 CUR-HSA-DOPE NPs 25.2 ± 5.8% and 11.9 ± 8.6% free CUR 34.5 ± 6.6% and 48.3 ± 7.2% 50 μg of curcumin	Immunofluorescence TUNEL assay ↑ Apotosis CUR-HSA-DOPE NPs	None
Kumari et al., 2016 [54] India	MDA-MB-231 Human	Curcumin at 50 μg/mL Curcumin and curcumin-loaded nanoparticles (curcumin in mPEG-PLA micelles) (CUR-HSA-DOPE NPs)	24 h	Not reported	MTT Assay CUR-mPEG-PLA231 35.1 ± 8.5 free CUR 65.7 ± 1.0% 50 μg/mL	Not reported	None
Laha et al., 2018 [55] India and USA	MDA-MB-468 Human	Curcumin at 20, 40, 60, 80, 100, and 120 mM	12 and 24 h	Not reported	Not reported	Annexin V-FITC staining Apoptotic cells: 25% and 91%.	None
Lai et al., 2012 [56] China	MCF-7, BT-474, MDA-MB-231, and normal breast cells Human	Curcumin at 10 μg/mL	72 h	Colorimetric analysis of sulforhodamine B ↓ cell proliferation (MCF-7, BT-474, and MDA-MB-231 cells).	Not reported	Not reported	None
Li et al., 2018 [3] China	MDA-MB-231 Human	Curcumin at 10 g/mL Nanostructured platform curcumin and curcumin nanoparticle MSN/IR780-PEI-FA 160 mg/kg	24 and 48 h	Not reported	Not reported	Flow cytometry CUR and free MSN/CUR induced the G2/M phase of the cell cycle.	
Liu et al., 2013 [58] China	4T1 Mouse	Curcumin at 100 μg/mL Nanostructured platform Nanoparticle with self-assembled polymeric micelles (CUR-M) loaded with curcumin (CUR)	48 h	Not reported	MTT assay Both CUR-M and Free CUR drastically inhibited cell growth in a dose-dependent manner.	TUNEL assay by immunofluorescence staining Apoptotic index CUR-M: 15.77 ± 2.74%, Free CUR: 9.42 ± 2.13% *p* < 0.001)	None
Liu et al., 2009 [58] China	MDA-MB-231 Human	Curcumin at 1, 1.25, 2.5, 5, 10, and 20 mg/mL	24 and 48 h	Method (NR) Inhibition of cell growth by 60–70% with 1.25 mg/mL curcumin. Inhibition of cell growth by 50–60% with 2.5 mg/mL curcumin.	Not reported	Not reported	NR
Lv et al., 2014 [61] China	MDA-MB-231 Human	Curcumin at 1–100 μL	24 and 48 h	Not reported	MTT assay ↓ significant reduction in the number of viable cells in a time- and dose-dependent manner.	Flow cytometry of fixed nuclei ↑ in the number of apoptotic cells in a dose-dependent manner.	None
Masuelli et al., 2013 [63] Italy	MDA-MB-231, MDA -MB-435, MDA-MB-453, MDA-MB-468, T-47D, MCF7, BT-474, SK-BR-3 Human Mammary cancer cells (H-2”) (TUBO) Humanized mouse Mammary cancer cells (H-2”) (TUBO) Mouse	Curcumin 6 to 50 pM	24 and 48 h	Not reported	Not reported	Pro-apoptotic Bax and anti-apoptotic Bcl-2 expression CUR induced apoptosis in all investigated cell types.	None
Mehta et al., 1997 [64] USA	MCF7 Human	Curcumin 1 to 3 μg/mL	72 h	[3H]thymidine incorporation and flow cytometry. Cell growth inhibition in a time- and dose-dependent manner, correlated with the inhibition of ornithine decarboxylase activity.	Not reported	Flow cytometry Curcumin-induced cell death was not due to apoptosis or any significant change in the expression of apoptosis-related genes, including the Bcl-2, p53, cyclin B, and transglutaminase genes.	NR
Montazeri et al., 2017 [65] Iran	MCF7 Human	Curcumin at 23, 17, and 14 µM Dendrosomal curcumin (DNC) for 48 h (28–35 μM) and 72 h (23–25 μM)	24, 48, and 72 h	Not reported	Not reported	Flow cytometry Total apoptosis by DNC: 24 h: 30.34 ± 0.011% 48 h: 33.83 ± 0.005% 72 h: 61.83 ± 0.009%	None
Mukhopadhyay et al., 2020 [67] India	MDA-MB-231 Human	5 mg of curcumin Nanostructured platform Polymeric NPs PLGA/PVA with or without folate (F)	24 h	Not reported	Not reported	Flow cytometry Apoptosis CUR-NP-F: 29% Free CUR: 20%	
Sarighieh et al., 2020 [70] Irã	MCF7 Human	Curcumin 5, 10, 20, 40, 80, and 160 μM	24 h	Not reported	MTT assay Curcumin decreased the cell viability of MCF-7 cells.	Flow cytometry Apoptosis 24.6%	None
Sun Shih-Han et al., 2012 [73] Taiwan	MDA-MB-231/Her2 Human	Curcumin at 30 and 50 mM	24 h	Not reported	Not reported	Flow cytometry Apoptosis occurred at a higher dosage (50 mM).	None
Sun Xiao-Dong et al., 2012 [74] China	MDA-MB-231 Human	Curcumin at 10, 20, and 30 μmol/mL	48 h	MTT assay The inhibitory effect on MDA-MB-231 cell proliferation peaked upon treatment with 30 μmol/mL curcumin (*p* < 0.01).	Not reported	Flow cytometry Apoptosis control 2.76% and Curcumin 26.34%, 30 μmol/mL (*p* < 0.01).	None
Wang Xet al., 2017 [76] China	MCF-7 Human	Curcumin [0 (with DMSO vehicle), 0.5, 1.0, 2.0, 5.0, and 10.0 µM]	24, 48, and 72 h	MTT assay ↓ cell growth (treatment with 0, 0.5, 1.0, 2.0, 5.0, and 10.0 µM curcumin).	Not reported	Flow cytometry Apoptotic cell death within 48 h upon treatment with 2 µM (*p* = 0.0021) and 5 µM (*p* = 0.0004) curcumin.	None
Yang et al., 2017 a [76] China	MCF-7 Human	Curcumin at 50 μm Nanostructured platform Micelle NPs (PPBV triblock copolymer)	24 h	Not reported	Not reported	Flow cytometry Apoptotic cell death	
Younesian et al., 2017 [80] Irã	SKBR3 Human	Curcumin at 2.5, 10, 15, 20, 25, and 30 μM	24, 48, and 72 h	Not reported	Not reported	Flow cytometry Apoptosis: 4.37% with 0 μM, 27.46% with 5 μM, 64.98% with 10 μM, 75.90% with 15 μM, and 76.92% with 20 μM curcumin.	None
Yu et al., 2021 [81] China	4T1 Mouse	Curcumin at 5, 10, and 15 μM	24 h	Not reported	MTT assay ↓ of cell viability by 16% using 15 μg/mL curcumin	Not reported	None
Zong et al., 2012 [84] China	MCF-7 Human	Curcumin at 10, 20, 50, and 100 μM	48 h	MTT assay ↓ cell growth by 37%, 54%, and 73% using 20, 50, and 100 μM curcumin, respectively.	Not reported	Not reported	None

MTT assay, MTT Assay Protocol for Cell Viability and Proliferation, ↓: inhibition, ↑: activation.

**Table 2:** Characteristics of the studies conducted on experimental animal models included in the systematic review on curcumin and breast cancer.

Author/Year/Country	Experimental Animal Model *	Intervention		Outcome	Conflicts of Interest	Ethical Approval
		Treatment Follow-Up	Dose (mg/kg)/Administration Route	Anti-Tumor Activity (Size or Volume of the Tumor)		
Abd-Ellatef et al., 2020 [38] Italy and Egypt	Balb/c/n = 8/JC/mouse/(1 × 10^7^ cells)/mammary fat pad	VT: 50 mm^3^; three times (on days 1, 7, and 14); vehicle-free CUR: 10% DMSO suspension *v*/*v* Follow-up: 18 days **Nanostructured platform** Solid lipid nanoparticles (SLNs) with or without chitosan (CS) coating (cholesterol; trilaurin, butyl lactate, Epikuron^®^ 200, Cremophor^®^ RH60, sodium taurocholate, Pluronic^®^ F68)	5 mg/kg; Intravenous administration	CURC-CS-SLN and CURC ↓ VT (35%); Free CUR: no VT ↓; *p* < 0.01	None	Yes
Alizadeh et al., 2015 [29] Iran	Balb/c/n = 8/Transplantation of spontaneous mouse mammary tumor/pieces < 0.3 cm^3^/subcutaneous administration in the left flank	14 days after tumor induction; daily for 24 days Follow-up: 35 days **Nanostructured platform** Micelles/polymersomes NPs (PNP) [monomethoxyPEG (mPEG 2000), oleic acid (OA)]	Dose: (NR); Intraperitoneal administration	CUR-NP ↓ VT (80%); *p* < 0.05	None	Yes
Bansal et al., 2014 [30] USA	Female ACI mice/ 5 to 6 weeks old/mammary tumorigenesis mediated by 17β-estradiol (E2)/9 mg of E2/back	4 days after tumor induction/ Curcumin implants (n = 6) Curcumin diet (n = 6) Follow-up: 6 months	Curcumin 1000 ppm via diet Two 2 cm implants, 200 mg/implant, 20% p/p drug load 10.9 mg of curcumin for 25 days subcutaneous administration	Curcumin implant: ↓ VT (35%) Curcumin administration via diet: ineffective	None	Yes
Bimonte et al., 2015 [7] Italy	Foxn1 nu/nu female mice/n = 16, 6-to-8-week-old/human breast cancer cell line MDA.MB231/2.5 × 10^6^ cells/right flank	After reaching 30–60 mm^3^, normal diet (n = 8) and diet containing 0.6% curcumin were administered (n = 8). Follow-up: 6 weeks	0.6% Curcumin administration via diet	↓ VT (% NR) (*p* = 0.0195)	None	NR
Chen et al., 2017 [32] China	Balb/c/n = 5/BT-549/human (2 × 10^6^ cells)/subcutaneous administration in the right upper thigh	200 mm^3^ VT 35 mg/kg; Fourteen days, every 2 days Intratumoral—Vehicle Free CUR: NR Follow-up: 30 days Nanostructured platform Micelle NPs [POCA4C6 (phosphorylated calixarene) micelles—PM]	5 mg/kg; Intratumoral administration	CUR-NP ↓ VT (60%); Free CUR: ↓ VT (34%); *p* < 0.05	None	Yes
Ghosh et al., 2021 [39] India	Swiss albino mice/3 groups (n = 5)/MCF-7 and MDA-MB 231 cells (human)/vein	Alternating days after tumor induction Follow-up: 2 weeks Nanostructured platform Nanoparticles: MSN-Curcumin (MSN-C) and MSN-Hyaluronic acid-Curcumin (MSN-HA-C)	10 mg/kg; intravenous administration	MSN-HA-C ↓ VT (% NR); *p* < 0.05	None	Yes
Greish et al., 2018 [40] Bahrain	Balb/c/n = 5/4T1/mouse/(1 × 10^6^ cells)/bilaterally on flanks	VT: 100 mm^3^; frequency of treatment: unclear; Treatment: 10 days Follow-up: 9 days Nanostructured platform Micelles (curcumin-metal complex and SMA)	10 and 20 mg/kg; Intravenous administration	CUR-NP-10 mg/kg ↓ VT (61%); CUR-NP-20 mg/kg ↓ VT (92%); *p* < 0.05	None	NR
Grill et al., 2018 [41] Estados Unidos	Balb-neuT mice/n = NR/HER-2-positive breast cancer cells/ten breast pads	At 2, 4, 7, or 12 weeks of age, and once a month thereafter Follow-up: 24 weeks **Nanostructured platform** Curcumin-loaded microparticles Curcumin (20 mg) and PLGA (20 mg)	140 mg of microparticles, corresponding to 58.2 mg of curcumin/administered via subcutaneous injection	Curcumin MP ↓ VT (60%); *p* < 0.05	None	Yes
Hashemzehi et al., 2018 [42] Iran	Balb mice/n = 4/MCF-7 cells (human)/flanks	VT: 100 mm^3^; 7 days after tumor induction Follow-up: 22 days Nanostructured platform Nanocurcumin—phytosomal curcumin	Dose: (NR); NR	Curcumin ↓ VT (22.2%) Curcumin + 5-FU ↓ VT (53.3%)	None	Yes
He et al., 2019 [43] China	Balb/c/n = 6/4T1/mouse/(1 × 10^6^ cells)/subcutaneous administration in right back	VT: 100 mm^3^ Every 4 days for 4 times Free CUR: (NM) Follow-up: 21 days Nanostructured platform Polymeric micellar NPs [amphiphilic diblock copolymer—mPEG-b-PLG (Se)-TP]	5 mg/kg; Intravenous administration	CUR-NP ↓ VT (62.9%); Free CUR: ↓ VT (55%); *p* < 0.05	None	Yes
Huang et al., 2020 [46] China	Balb/c/n = 5/4T1/mouse/NR/Flank mice	VT: 40–50 mm^3^/every 2 days for 5 times Follow-up: 16 days **Nanostructured platform**	50 mg/kg; Intravenous	CUR-NP ↓ VT (38%); *p* < 0.05	None	Yes
Ji et al., 2020 [47] China	Balb/c/n = 5/4T1/mouse/(1 × 10^6^ cells)/subcutaneous administration in the right flank	Polymeric NPs (HA-CHEMS); pH-sensitive First day of treatment: NR; Every 2 days Vehicle-free CUR: (NM) Follow-up: 10 days **Nanostructured platform** Nanocrystal NPs with or without HA	5 mg/kg; Intravenous	HA@CUR-NCs ↓ VT (86%); CUR-NP ↓ VT (39%); Free CUR: ↓ VT (21%); *p* < 0.05	None	Yes
Jin et al., 2017 [49] China and USA	Balb/c nude rats/n = 5/MCF-7/human/(1 × 10^7^ cells)/subcutaneous administration in the dorsal flank	7 days after tumor induction; every 24 h for 20 times Free CUR: NR Follow-up: 3 weeks Nanostructured platform Polymeric NPs with or without EGFR-targeting peptides (GE11) (PLGA-PEG);	5 mg/kg; Intravenous administration	CUR-NP-GE11 and CUR-NP ↓ VT (80%); Free CUR: sem VT ↓; *p* < 0.05	None	Yes
Jung et al., 2018 [50] República da Coréia	Balb/c nude rats/n = 4/MDA-MB-468 cells/human/(5 × 10^6^ cells)/right shoulder	**VT: 50 mm^3^; three times a week; eight injections in all** Follow-up: NR **Nanostructured platform** CUR-NP e EGF-CUR-NP	10 mg/kg; Intraperitoneal administration	CUR-NP-EGFR ↓ VT (59.1%); CUR-NP no ↓ VT; *p* < 0.05	None	Yes
Kumari et al., 2020 [52] India	Balb/c mice/n = 18/Mouse (4T1)/50 μL, 1 × 10^6^ cells/subcutaneous administration in left flank	VT: 50 mm^3^; Follow-up: 21 days **Nanostructured platform** CUR treatment (Free CUR group (0–24 μg/mL)) and CUR-HSA-DOPE NPs treatment (CUR-HSA-DOPE group)	25 mg/kg; Intravenous administration	CUR-HSA-DOPE ↑ VT (80.41%); Free CUR ↑ VT (86.30%)	None	Yes
Laha et al., 2018 [55] India and USA	Balb/c/n = 6/4T1/mouse/NR/mammary fat pad	10 days after tumor induction; every 5 days for four times Follow-up: 20 days **Nanostructured platform** Metal organic frameworks NPs (IRMOF-3) with or without folic acid (FA) [(Zn(NO_3_)_2_; NH_2_-H_2_ BDC]	2 mg/kg (* unclear); Route of administration: (NM)	CUR-NP-FA ↓ VT (61%); CUR-NP ↓ VT (44%); *p* < 0.05	None	Yes
Lai et al., 2012 [56] Taiwan	Nude mice/n = 16/BT-474 cells overexpressing HER-2 (1 × 10^7^)/right flank subcutaneous route of administration	21–28 days after xenograft inoculation. VT:50–100 mm^3^ Follow-up: after 4 weeks	45 mg/kg curcumin injected intra-peritoneally	Herceptin and curcumin VT 34.1 ± 25.0 mm^3^ Curcumin VT 63.6 ± 25.7 mm^3^ *p* = 0.079		
Li et al., 2018 [3] China	Balb/c/n = 4/MDA-MB-231/human/(1 × 10^7^ cells)/subcutaneous administration	Tumor diameter: 4 mm; every 3 days for six times in all Free CUR: NR Follow-up: 18 days Nanostructured platform Mesoporous silica nanoparticles with hyaluronan (MSN-HA) or polyethyleneimine-folic acid (MSN-PEI-FA).	8 mg/kg; Intravenous administration	CUR-NP-PEI-HA ↓ VT (50%); Free CUR: no VT ↓; *p* < 0.01	None	Yes
Lin et al., 2016 [57] China	Balb/c nude mice/n = 6/MCF-7/ human/(NM)/Subcutaneous administration in the right axilla	First day of treatment: NR once every 3 days for 15 days Vehicle-Free CUR: (NM) Follow-up: 15 days **Nanostructured platform** Lipid-based NPs (NLC) with or without folate coating (FA) (PEG-DSPE, soy lecithin, castor oil, Tween 80, and Precirol ATO-5)	Dose: NR; Intravenous administration	CUR-NP-FA ↓ VT (~83%); CUR-NP ↓ VT (~66%); Free CUR: ↓ VT (31%)	None	NR
Liu et al., 2013 [58] China	Balb/c mice n = 12; 6 per group/4T1/5 × 10^5^ cells/right flank/subcutaneous administration	From day 4, palpable tumors were daily injected with the treatment agent intravenously for 10 days Follow-up: 25 days Nanostructured platform Self-assembled polymeric micelles (CUR-M) loaded with curcumin (CUR)	CUR-M (30 mg/kg body weight) Free CUR (30 mg/kg body weight)	CUR-M ↓ VT (68%); *p* < 0.01 Free CUR: sem ↓ VT (35%)	None	Yes
Lv et al., 2014 [61] China	Balb/c nude mice/n = 8 per group /MCF-7 and MDA-MB-231/2 × 10^6^ cells/subcutaneous administration in the back	After reaching 60 mm^3^/treatment days alternating Follow-up: 4 weeks	Curcumin 50 µg/kg, 200 µg/kg Intraperitoneal injections	Cur 50 µg/kg ↓ VT (54%); *p* < 0.05 Cur 200 µg/kg ↓ (73%); *p* < 0.05 VT	None	Yes
Lv et al., 2015 [60] China	Kunming mice/n = 6/EMT6/mouse/(1.0 × 10^7^ cells/mL)/Subcutaneous administration	VT: 300 mm^3^; daily for 9 days Vehicle-free CUR: cremophor EL:dehydrated alcohol (1:1, *v*/*v*) and diluted with saline solution Follow-up: 14 days Nanostructured platform Polymeric NPs (PEG-PCDA) with or without biotin	10 mg/kg; Intravenous administration	CUR-NP ↓ VT (69%); CUR-NP-biotin ↓ VT (79%); Free CUR: ↓ TV (32%); *p* < 0.05	NR	Yes
Mahalunkar et al., 2019 [62] India, Germany and Norway	Balb/c/n = 6/4T1/mouse/(1 × 10^5^ cells)/Mammary fat pad	First day of treatment: (NM) Twice a week for 2 weeks Vehicle-free CUR: (NM) Follow-up: 21 days Nanostructured platform Metallic gold NPs (CurAu-PVP) with folic acid (FA) (HAuCl_4_ and PVP polymer)	10 mg/kg; Intratumoral administration	CUR-NP-FA ↓ VT (51%); Free CUR: no ↓ VT; *p* < 0.006	None	Yes
Masuelli et al., 2013 [63] Italy	Transgenic BALB-neuT mouse/n = 5 per group /NR	After the diameter reached 15 mm, CUR (2 mg in 50 |.il oil with), with oil (50 |.il) or water (50 |.il) was administered three times a week. Follow-up: 30 weeks	Curcumin 6–50 µM Oral administration	No mice treated with CUR exhibited tumor growth at week 22, (*p* < 0.01). Cur ↓ VT (52%) (*p* < 0.05)	None	NR
Mukerjee et al., 2016 [66] USA	Balb/c nude rats/n = 8/MCF10CA1a/human/(3 × 10^6^ cells)/flank	VT: 70 mm^3^; Three times a week for 30 days Follow-up: 32 days Nanostructured platform Polymeric NPs [PLGA/PVA with or without antibody targeting (AnxA2)]	20 mg/kg; Intravenous administration	CUR-NP-AnxA2 ↓ VT (44.0%); CUR-NP ↓ VT (33.5%); *p* < 0.05 CUR-NP-AnxA2 ↓ PT (53.0%); CUR-NP ↓ PT (30%); *p* < 0.05	NR	NR
Mukhopadhyay et al., 2020 [67] India	Balb/c nude rat/n = 5/MDA-MB-231/human/(5 × 10^6^ cells)/Right flank	8 days after induction; three times a week Follow-up: 29 days **Nanostructured platform** Polymeric NPs [PLGA/PVA with or without folate (F)]	20 mg/kg Route of administration: unclear	CUR-NP-F ↓ VT (90%); CUR-NP ↓ VT (75%); *p* < 0.05	NR	Yes
Pal et al., 2019 [68] India	Balb/c mice/n = 5 per group /human MCF-7, MDA-MB-231, MDA-MB-468, and murine 4T1/100 L/abdominal skin	Treatment for 20 days at 3-day intervals after 10 days of tumor implantation Follow-up: 30 days **Nanostructured platform** Synthesis of curcumin-loaded microsphere (10% by weight polymer) PLGA@CCM@FA	2000 µg/kg Route of administration: unclear	**PLGA**—VT 0.092 mm^3^ ↓ VT (25%) **PLGA @ CCM**—VT 0.064 mm^3^ ↓ VT (48%) **PLGA @ CCM @FA**-VT—0.031 mm^3^ ↓ VT (75%)	NR	NR
Sahne et al., 2019 [69] Irã	Balb/c/n = 4/4T1/mouse/NR/ssubcutaneous administration in the flank	VT: 50–100 mm^3^; daily follow-up: 3 weeks **Nanostructured platform** Graphene oxide NPs (GO NPs with CMC, PVP, PEG, and FA)	4 mg/kg; Intravenous administration	CUR-NP-FA ↓ VT (86%); *p* < 0.05	None	Yes
Shiri et al., 2015 [71] Irã	Balb/c/n = 9/4T1/mouse/(1 × 10^6^ cells)/left flank	Third day after tumor induction Follow-up: 35 days **Nanostructured platform** Dendrosome NPs (DNC) [composition: not mentioned (patent number: 71753)].	40 or 80 mg/kg Route of administration: NR	NP-40 mg/kg ↓ VT (72%); NP-80 mg/kg ↓ VT (76%); *p* < 0.05 NP-40 mg/kg ↓ VT (61%); NP-80 mg/kg ↓ VT (64%); *p* < 0.05	NR	Yes
Shukla et al., 2017 [72] India	Balb/c mice/n = 3/(1 × 10^6^ cells)/subcutaneous administration in hind skin	10 days from tumor inoculation; daily administration for 28 days: gum acacia (1%, *w*/*v*). Follow-up: 42 days **Nanostructured platform** Lipid-based CPC-SNEDDS NPs (Phospholipid, castor oil, Tween 80, and PEG 400)	100 mg/kg; oral	1) CUR-NP ↓ VT (58.9%); Free CUR ↓ VT (29.5%); *p* < 0.001	None	Yes
Vakilinezhad et al., 2019 [75] Irã	Sprague–Dawley rats/n = 6/Chemically-induced mammary tumors (MNU)	4 months after tumor induction; Once a week for 4 weeks Free curing vehicle: aqueous suspension Follow-up: 20 weeks **Nanostructured platform** Polymeric NPs (PLGA-PVA)	2.5 mg; Intravenous	CUR-NP ↓ VT (20%); Free CUR: ↓ VT (16%); *p* < 0.05	None	Yes
Wang et al., 2018 [77] China	Nude mice/n = (NM)/MDA-MB-231/human/(1.5 × 10^6^ cells)/subcutaneous	2 months after tumor induction; daily Free CUR: (NM) Follow-up: 2 weeks **Nanostructured platform** Polymeric NPs (MPEG-PCL)	1 × 10^−3^ M; Intravenous administration	CUR-NP ↓ VT (82%); Free CUR: ↓ VT (49%); *p* < 0.01	None	Yes
Yang et al., 2017 a [78] China	Balb/c nude mice/n = 5 MCF-7/human/(1 × 10^7^ cells)/subcutaneous administration in the flank	VT: 200 mm^3^ Every other day, five times; total duration: 20 days Free CUR vehicle: NR Follow-up: 20 days **Nanostructured platform** Hybrid NPs [PLGA NPs coated with a modified hyaluronic acid (HA hybrid)]	15 mg/kg; Intravenous	HA-Hybrid NPs/CUR ↓ VT (43.8%, day 12); ↓ VT (24%, day 20); *p* < 0.05	NR	Yes
Yang et al., 2017 b [79] China	Balb/c nude mice/n = 5 MCF-7/human/(1 × 10^7^ cells)/subcutaneous administration in the flank	VT: 200 mm^3^ Every other day, five times; total duration: 20 days Free CUR vehicle: NR Follow-up: 20 days Nanostructured platform Micelle NPs (PPBV triblock copolymer)	10 mg/kg; Intravenous	PPBV micelles/CUR ↓ VT (58.5%, day 12); ↓ VT (28.9%, day 20); *p* < 0.05	NR	Yes
Yu et al., 2014 [82] China	Balb/c nude mice/n = 5/MCF-7/human/(3 × 10^6^ cells)/subcutaneous administration in the right flank	VT: 100–400 mm^3^; Every other day for 5 times for 24 days in all Follow-up: 25 days **Nanostructured platform** Micelle NPs (MPEG-PLA with or without PAE)	40 mg/kg; Intravenous administration	CUR-NP-PAE ↓ VT (65.6%); CUR-NP ↓ VT (47.1%); *p* < 0.05	NR	Yes
Yu et al., 2021 [81] China	Balb/c mice/ murine 4T1/NR/intradermal administration in the back of the neck	VT: 150–200 mm^3^, administration via tail vein every 3 days; 14 days in all Follow-up: 16 days **Nanostructured platform** curcumin (CUR), zeolitic imidazolate framework-8 nanoparticles (ZIF-8), and hyaluronic acid (HA)	CUR@ZIF-8 19.6 mg of CUR@ZIF-8@ HA 20.9 mg Intravenous administration	CUR@ZIF-8 ↓ VT (12.5%); CUR@ZIF-8@HA ↓ VT (62.5%);	None	Yes
Yuan et al., 2018 [83] China	Balb/c nude mice/n = 6/MCF-7/human/(3 × 10^6^ cells)/right flank	VT: 100 mm^3^; every other day, four times Follow-up: 18 days **Nanostructured platform** Polymeric NPs (mPEG-PLGA-Pglu)	2.5 mg/kg; intravenous administration	CUR-NP ↓ VT (28.0%); *p* < 0.05 CUR-NP ↓ PT (22.5%); *p* < 0.05	None	Yes

* Animal type/sample size/injected cell type/source/cell concentration/cell insertion site; NR: not reported; VT, tumor volume; ↓: inhibition; ↑: activation.

#### 3.2.3. Cell Viability

Cytotoxicity in breast cancer cell lineages was assessed using the MTT assay. Curcumin significantly decreased the viability of MCF-7 malignant cells in a time and dose-dependent manner [27,49,70]. In another trial, a decrease in the viability of MCF-7 cells by 49% and of MDA-MB-453 cell cultures by 48% following treatment with 20 µM curcumin for 24 h was observed [36], while another study reported that curcumin did not affect the viability of MCF-7 cell cultures [51]. Cells were treated with 1, 5, 10, 30, and 50 μM curcumin for 24 h at 37 °C.

There was a significant decrease in the viability of MDA-MB-468 cells upon treatment with 10 µM curcumin [50]. The viability of triple-negative MDA-MB-231 cell cultures reduced by up to 25% upon treatment with 15–100 μM curcumin for 24 h [28,37,53,54,61]. There was a 55.2% reduction in the viability of MDA-MB-23 colonies upon treatment with 50 μg/mL curcumin [52]. In T47D cell lineages, viability reduced by 48% and 60% upon treatment with 20 µM curcumin [35].

Mouse 4T1 cultures showed a significant reduction in cell viability upon treatment with pure 6–50 pM curcumin [43,53,81]. Curcumin CUR-M and free CUR nanoparticles also inhibited cell growth in a dose-dependent manner [58].

#### 3.2.4. Apoptosis and/or Interruption of Cell Cycle

In most studies, apoptosis and/or interruption of the cell cycle were assessed using the MTT assay [28,36,37,39], quantitative image analysis [34], Annexin-V/PI double staining [35,48], immunofluorescence TUNEL assay [53,58], Annexin V-FITC staining [55], pro-apoptotic Bax and anti-apoptotic Bcl-2 expression evaluation [63], and flow cytometry. 

In the breast cancer MCF-7 cell lineage, apoptosis occurred in 4.6% of the cells upon treatment with 25 μM curcumin [34], and in 28.7% and 49% of the cells upon treatment with 30 μM curcumin [31,36,48]. Other studies reported 14.9% apoptosis in MCF-7 colonies treated with 10 µg/mL curcumin delivered via nanoparticles [49]. There was 24.6% apoptosis in MCF-7 cells incubated under normoxic and hypoxic conditions for 24 h and treated with curcumin at different concentrations (5, 10, 20, 40, 80, and 160 μM) [70]. Wang et al. also reported apoptosis following treatment with 2 and 5 µM curcumin for 48 h [76]. 

In the triple-negative MDA MB-468 cell lineage, the apoptosis frequency was 25% and 91% after treatment for 12 and 24 h, respectively [55]. In addition, 30 μM curcumin induced apoptosis in 31.36% of MCF-7/LCC2 cells [48] and 34.70% of LCC9 cells [48]. Other studies also reported apoptosis in triple-negative MDA-MB-231 cells treated with 10, 12, 20, 30, and 50 μM curcumin for 24 and 48 h [3,7,28,31,33,37,39,47,53,61,67,74], and in colonies of SK-BR-3 cells treated with 5, 10, 15, and 20 μM curcumin [63,80]. In T47D cells, 30 µM curcumin induced 10.9% apoptosis in 24 h, 5.2% apoptosis in 48 h [35], and 30.09% apoptosis in 72 h [44].

Mouse 4T1 cell lines showed increased apoptosis in response to treatment with 6 to 50 pM curcumin [43,53,63]. Moreover, CUR-NPs at 0–100 μg/mL also induced apoptosis in a dose-responsive manner [58].

#### 3.2.5. Animal Studies 

Thirty-seven studies on animal models met the inclusion criteria [3,7,29,30,32,38,39,40,41,42,43,46,47,49,50,53,55,56,57,58,60,61,62,63,66,67,68,69,71,72,75,77,78,79,81,82,83]. Curcumin was delivered via diet in two studies [7,63], diet and implant in one study [30], intraperitoneal injection in two studies [56,61], and different modes using nanoparticles in 32 studies. The results of these studies are listed in Table 2, with the animal species, sampling size, type of cells injected, cell concentration, cell insertion site, treatment, follow-up, dose, and route of administration specified. The studies were heterogeneous with respect to the animal model, follow-up, curcumin dose, and route of administration. 

A curcumin-encapsulated polymer micelle formulation was developed showing antitumor and anti-metastatic activities in breast cancer cells [58]. Micelles loaded with curcumin inhibited tumor activity and induced minimal collateral effects in vivo compared to a free curcumin formulation (free-CUR) [43]. Reduction in tumor volume increased significantly following treatment with CUR-NPs (20–92%) [29,32,38,40,46,57,58,60,67,72,75,76,83] rather than with free CUR (0–55%) [32,38,40,43,47,49,60,62,72,75,77,83].

Other curcumin delivery methods and their corresponding tumor magnitude percentile reductions were as follows: HA@CUR-NCs (86%) [47], CUR-NP-biotin (79%) [60], curcumin + 5-FU (53.3%) [42], CUR-NP- AnxA2 (44.0%) [66], CUR-NP-PEI-HA (50%) [3], HA-Hybrid NPs/CUR (43.8%) [78], PPBV micelles/CUR (58.5%) [79], CUR-NP-FA (51–86%) [57,62,69], CUR-NP-PAE (65.6%) [82], CUR@ZIF-8@HA (62.5%) [81], and CUR-NP-EGFR (59.1%) [55]. Furthermore, the synthesized nano-hybrid MSN-HA-C increased anticancer efficacy when compared to Free CUR [39].

Intracellularly degradable, self-assembled amphiphilic biotin-poly (ethylene glycol)-b-poly (curcumin–dithiodipropionic acid) nanoparticles exhibited excellent anticancer activity in vivo due to their high tumor-targeted accumulation and stimuli-triggered intracellular drug release [60]. Moreover, these nanoparticles could be loaded with other anticancer drugs, which could promote synergistic oncologic effects in vivo [60]. 

In another trial, compared to control PLGA microparticles, curcumin-loaded microparticles retarded oncogenesis in a Balb-neuT transgenic mouse model. PLGA microparticles accelerated oncogenesis compared to a saline control. This unanticipated collateral effect of PLGA microparticles may be related to the high dose of microparticles for optimal in vivo concentration of curcumin [41].

### 3.3. Conflict of Interest and Ethics Committee Approval

Only six studies were approved by their respective ethics committees on animal use [7,40,57,63,66,68], while there was no mention of potential conflicts of interest in eight studies [60,66,67,68,71,78,79,82]. The authors of the remaining articles declared no conflicts of interest.

### 3.4. Overall Quality of Evidence

Thirty-nine studies were rated as moderate with respect to quality of evidence using the GRADE approach [25], as shown in (Table S3). These studies were not representative of the results of all assessed outcomes. 

The evaluation of the risk of bias based on the SYRCLE RoB Toll guidelines for animal model studies is shown in Table 3. Most studies did not clearly state information on assignment, randomization, and blinding, which are critical aspects for assessing the quality of evidence.

## 4. Discussion

This systematic review highlighted some of the promising antitumor activities of curcumin reported in in vitro studies, as well as its potential for tumor volume reduction in animal models. At different concentrations, curcumin inhibited cell proliferation, reduced cell viability, and induced apoptosis in several human and animal breast cancer cell subtypes. In vivo data showed that curcumin reduced tumor volume in human and murine mammary cells when administered either orally, via implants, or via intraperitoneal injection, or when delivered via different curcumin nanoparticle formulations. 

In vitro studies showed inhibitory activity of curcumin on cell proliferation, induction of cell viability, and apoptosis at different concentrations. The anti-proliferative effect of curcumin was attributed to its regulatory effects on protein kinases, the cell cycle, and transcription factors, including NF-κB [85]. Curcumin significantly inhibited the growth of MDA-MB-231 and MCF-7 human breast cancer cells by inducing apoptosis in a gradual, dose-dependent method, which was related the increase in the Bax/Bcl-2 ratio [34,61].

**Figure 2:**
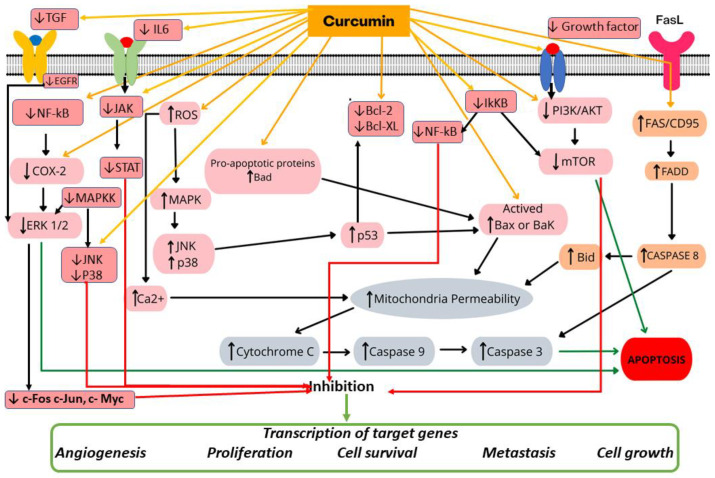
Cellular and molecular mechanisms of action of curcumin in breast cancer. Curcumin exerts its anticancer effect by modulating cell proliferation, inducing apoptosis and inhibiting cancer spread. JAK: janus kinase, STAT: signal transducer and activator of transcription, IL-6: interleukin-6, IκKB: inhibitor of kappa B kinase, TGF: transforming growth factor, EGFR: epidermal growth factor receptor, MAPK: mitogen-activated protein kinase, MAPKK: MAPK kinase, JNK: c-Jun N-terminal kinases, Bcl-2: B-cell lymphoma 2, Bak: Bcl-2 homologous antagonist/killer, Bad: BCL2 associated agonist of cell death, Bid: BH3 interacting-domain death agonist, Bax: Bcl-2 associated X protein, Bcl-xL: ROS: reactive oxygen species, NF-κB: nuclear factor-κ-gene binding, COX-2: Cyclooxygenase 2, ERK1/2: extracellular regulated protein kinase 1 and 2, PI3K: phosphatidylinositol 3-kinase, Akt: protein kinase B, mTOR: mammalian target of rapamycin, JNK: Jun N-terminal kinase, FADD: Fas-associated protein with death domain, p38: mitogen-activated protein kinases, FAZ/CD95: type-II transmembrane protein that belongs to the tumor necrosis fator, Caspases: cysteine-dependent aspartate-specific protease, p53: tumor-suppressor protein, ↓: inhibition, ↑: activation.

The cell cycle is divided into four phases: G1, S, G2, and M [85]. Dendrosomal curcumin increases the number of cells in the SubG1 phase and reduces the number of cells in the G1, S, and G2/M phases [65]. Early-stage apoptosis showed the inhibition of cell growth through the early phase. Real-time PCR revealed a gradual increase in the mRNA levels of BAX, NOXA, and p21, with a decrease in Bcl-2 expression [65]. The magnitude of anticancer effects and induction of apoptosis are essential for investigating antineoplastic therapy. Apoptosis occurred via intrinsic or mitochondrial pathways [85]. Apoptotic pathways were modulated via NF-κB and Bax [39,67]. Curcumin was also shown to downregulate the expression of cyclin D1, PECAM-1, and p65, which are regulated by NF-κB [7,35]. Figure 2 shows different mechanisms of action of curcumin in breast cancer, including cell proliferation, cell viability, and apoptosis.

The PLGA@CCM@FA nanoparticle formulation triggered apoptosis in human triple-negative breast cancer cells by positively regulating cleaved caspase-3 and downregulating p-AKT expression [68]. Curcumin also induced caspase-mediated apoptosis by activating the expression of polyamine catabolic enzymes, with the subsequent generation of toxic molecules such as H_2_O_2_ in MCF-7, MDA-MB-453, and MDA-MB-231 GH+ breast cancer cells [35]. Curcumin-encapsulated polymeric micelles should be considered for breast cancer treatment, as they reduced the proliferation of breast cancer cells [58]. Curcumin-loaded micelles also showed significant tumor-inhibiting properties as well as minimal in vivo collateral effects compared to free-CUR formulations [43]. A study revealed that alginate–chitosan hydrogel loaded with curcumin significantly reduced the viability and induced the apoptosis of malignant cells. Therefore, this system presents promising anticancer drug delivery properties [86].

Conversely, one of the pharmacological limitations of orally administered curcumin is its low bioavailability owing to its low solubility in water and rapid metabolism, which may hinder its clinical application [72,87]. In a randomized clinical study, water-soluble injection formulations of curcumin for parenteral/intravenous administration showed up to 100% bioavailability, demonstrating its potential clinical application [87]. Moreover, a liquid droplet nanomicellar formulation containing Gelucire^®^ and polysorbate 20 (BioCurc^®^) showed optimal bioavailability, with more than 400-fold greater absorption than non-formulated curcumin [88].

This study highlights different curcumin nanoparticle formulations with optimal bioavailability, causing substantial mammary tumor-reducing effects. Recent advances in micro-and nanoformulations of curcumin with enhanced absorption yield helped improve the serum levels of the active components. These formulations have a wide range of potential applications and properties, including tissue protection [89]. 

The results discussed in this review support randomized clinical investigations of the antitumor properties of curcumin in patients with breast cancer. Considering the diversity and heterogeneity of breast cancer subtypes, further studies will provide deeper insights into the effects of curcumin on specific types of mammary neoplasms to determine the effects on tumor markers, metastasis, and patient outcomes. Moreover, the efficiency and safety of curcumin in combination with other chemotherapeutic drugs should be established. In future clinical trials, tumor characteristics should be considered to support clinical decision-making. Both human patients and animals showing mammary neoplasms may benefit from curcumin-based therapies in the near future, as indicated by evidence from studies on animal models.

Although eight ongoing clinical assays on the effects of curcumin on breast cancer have been registered on clinicaltrials.gov to date, to the best of our knowledge, only one randomized controlled double-blinded clinical trial has been published [87]. In the said study, 150 women with advanced metastatic breast cancer were randomly assigned to receive paclitaxel chemotherapy (80 mg/m^2^) plus placebo or paclitaxel with curcumin (CUC-1^®^ solution, 300 mg, administered intravenously once a week) for 12 weeks, with three months of follow-up. The paclitaxel–curcumin combination provided a superior objective response and physical performance after two weeks of treatment. Intravenous curcumin was safe, did not negatively affect the patients’ quality of life, and decreased fatigue [87]. 

Currently, the data available only pertain to a trial at an advanced stage; therefore, studies focusing on early stages and, in particular, net-adjuvant chemotherapy are lacking. Addressing this knowledge gap remains essential. There are good prospects for the use of curcumin in cancer management, although its clinical development is limited due to its low bioavailability and aqueous solubility [90]; however, efforts have been made to improve the solubility, stability, and bioavailability of curcumin. For example, one strategy employed to obtain curcumin derivatives is chemical modification or synthesis of their analogues. Furthermore, curcumin encapsulated in protein nanoparticles demonstrated improved anticancer activity in MCF-7 cells and increased oral bioavailability in rats [90]. 

A systematic review [91] indicated that curcumin reduces the side effects of chemotherapy or radiotherapy, thereby improving the quality of life for patients. Furthermore, the authors reported that curcumin increases patient survival and decreases the level of tumour markers through several molecular pathways including hypoxic stress, angiogenesis, adhesion molecules, and extracellular matrix degradation [91].

Another review highlighted curcumin’s ability to interrupt important stages of tumorigenesis, including proliferation, survival, angiogenesis, and metastasis, in hormone-independent breast cancer, via the modulation of multiple signaling paths. The anticancer activity of curcumin in breast cancer was associated with the PI3K/Akt/mTOR, JAK/STAT, MAPK, NF-ĸB, p53, and Wnt/β-catenin pathways, as well as the apoptosis and cell cycle paths [9].

This systematic review provided a thorough overview of evidence from in vitro and animal model studies on the antitumor effects of curcumin in breast cancer. Our investigation was based on analysis of the five most important databases, with no restrictions imposed on the year of publication and language in the inclusion criteria. We included studies conducted in several countries, including China, India, Turkey, Iran, Italy, USA, Taiwan, Egypt, Bahrain, Romania, and Chile, which helped provide a broad perspective of the topic. However, this review had certain limitations. First, a meta-analysis could not be performed because of the high heterogeneity in the presentation of outcome measures, including different dosing and modes of delivery of curcumin, animal models, and methods of follow-up in the different studies. Furthermore, the adverse effects of curcumin formulations are yet to be investigated thoroughly. As the review did not focus on this aspect, we emphasize the importance of further studies investigating the adverse effects, toxicity, safety, tumor markers, and therapeutic responses in experimental trials and studies conducted on human patients. We believe that the results of ongoing clinical assays will provide a deeper understanding of the therapeutic potential of curcumin as an efficient alternative or adjuvant treatment.

## 5. Conclusions

This systematic review highlighted the beneficial effects of curcumin against human and animal breast cancer cells with respect to the inhibition of cell proliferation, reduction of malignant cell viability, and induction of apoptosis, and discussed the efficacy of curcumin in tumor growth reduction in experimental breast cancer models. These results were obtained from studies based on the delivery of curcumin via oral administration, implantation, intraperitoneal injection, and nanoparticle formulations. The information presented herein supports randomized clinical trials on the adjuvant properties of curcumin in the treatment of breast mammary neoplasms.

## Figures and Tables

**Table 3:** Risk of bias according to the SYRCLE’s RoB Toll criteria for animal models.

Authors	Selection Bias			Performance Bias		Detection Bias		Attrition Bias	Reporting Bias	Other Biases
	1	2	3	4	5	6	7	8	9	10
Abd-Ellatef et al., 2020 [38]	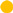	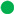	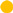	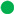	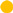	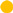	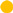	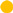	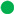	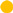
Alizadeh et al., 2015 [29]	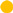	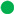	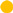	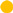	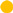	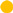	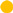	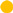	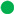	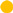
Bansal et al., 2014 [30]	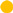	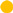	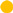	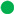	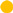	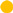	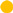	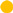	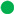	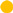
Bimonte et al., 2015 [7]	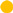	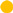	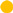	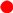	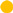	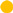	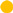	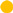	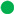	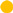
Chen et al., 2017 [32]	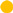	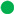	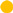	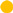	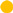	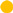	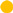	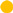	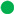	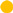
Ghosh et al., 2021 [39]	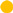	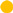	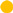	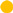	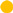	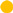	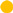	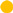	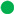	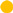
Greish et al., 2018 [40]	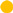	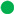	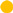	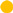	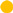	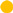	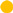	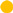	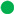	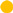
Grill et al., 2018 [41]	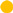	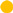	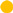	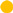	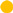	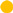	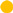	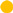	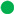	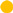
Hashemzehi et al., 2018 [42]	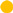	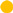	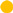	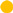	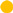	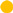	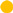	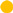	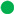	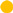
He et al., 2019 [43]	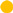	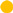	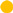	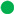	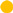	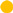	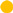	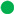	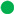	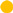
Huang et al., 2020 [46] China	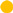	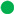	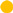	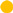	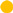	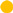	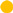	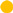	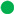	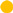
Ji et al., 2020 [47]	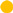	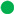	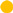	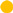	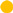	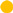	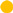	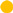	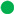	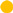
Jin et al., 2017 [49]	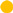	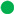	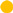	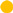	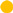	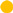	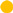	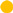	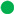	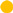
Jung et al., 2018 [50]	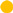	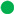	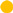	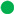	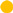	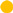	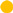	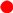	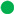	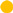
Kumari et al., 2020 [52]	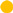	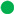	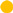	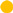	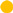	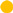	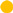	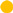	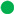	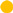
Laha et al., 2018 [55]	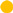	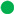	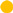	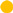	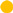	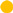	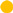	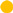	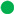	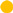
Lai et al., 2012 [56]	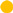	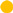	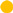	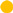	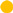	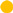	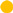	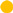	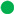	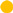
Li et al., 2018 [3]	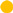	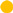	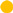	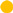	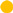	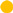	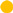	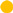	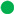	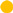
Lin et al., 2016 [57]	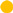	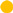	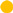	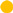	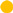	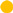	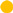	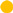	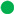	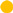
Liu et al., 2013 [58]	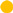	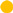	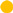	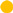	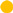	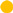	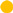	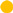	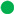	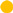
Lv et al., 2014 [61]	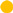	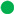	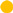	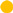	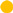	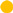	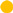	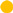	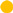	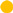
Lv et al., 2015 [60]	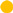	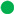	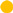	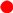	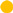	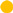	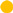	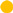	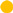	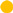
Mahalunkar et al., 2019 [62]	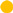	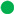	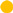	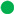	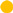	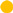	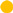	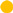	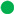	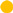
Masuelli et al., 2013 [63]	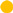	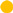	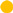	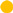	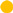	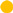	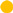	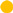	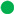	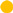
Mukerjee et al., 2016 [66] USA	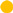	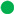	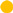	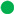	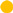	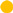	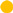	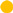	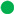	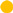
Mukhopadhyay et al., 2020 [67]	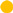	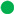	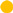	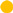	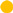	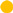	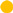	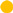	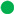	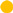
Pal et al., 2019 [68]	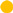	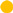	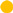	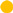	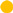	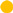	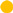	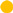	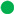	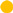
Sahne et al., 2019 [69]	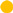	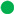	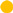	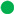	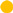	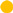	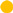	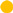	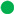	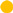
Shiri et al., 2015 [71]	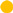	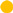	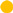	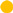	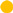	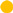	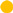	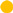	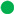	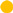
Shukla et al., 2017 [72]	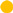	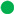	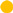	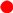	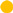	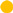	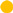	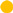	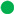	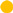
Vakilinezhad et al., 2019 [75]	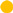	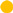	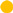	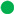	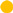	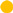	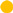	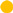	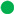	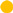
Wang et al., 2018 [77]	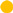	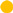	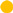	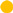	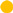	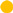	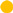	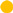	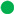	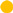
Yang et al., 2017 a [78]	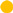	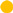	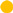	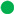	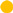	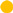	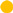	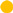	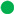	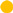
Yang et al., 2017 b [79]	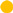	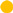	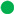	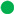	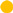	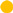	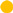	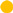	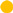	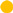
Yu et al., 2014 [82]	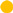	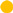	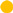	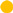	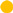	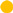	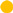	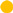	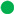	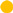
Yu et al., 2021 [81]	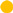	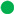	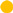	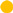	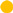	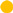	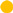	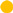	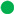	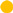
Yuan et al., 2018 [83]	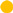	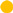	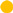	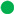	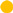	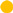	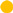	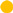	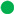	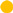

YES 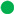 NO 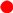 UNCLEAR 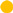. YES indicates low risk of bias; NO indicates high risk of bias; UNCLEAR indicates inability of bias assignment. The ten items assessed included: 1 Was the sequence of assignment generated and applied properly? 2 Were the groups similar at baseline, or were they adjusted for confounders in the analysis? 3 Was the allocation to the different groups adequately concealed? 4 Were the animals randomly housed during the experiment? 5 Were caregivers and/or investigators blinded to the intervention each animal received during the experiment? 6 Were the animals randomly selected for the evaluation of results? 7 Was the outcome assessor blinded? 8 Were data of incomplete results handled appropriately? 9 Are study reports exempt from selective result reporting? 10 Was the study apparently free from other problems that could cause a high risk of bias?

## Supplementary Materials

The following supporting information can be downloaded at: https://www.mdpi.com/article/10.3390/cancers14092165/s1, Table S1: Search strategies for use in the databases. Table S2: Articles excluded and reasons for exclusion. Table S3: Quality of evidence in the preclinical studies.
