# Supplementary material for: Antitumor Properties of Curcumin in Breast Cancer Based on Preclinical Studies: A Systematic Review
Source: Cancers (Basel). 2022 Apr 26;14(9):2165. doi: 10.3390/cancers14092165 (PMC9099919; doi:10.3390/cancers14092165)
Supplement: Supplementary file 1 [file cancers-14-02165-s001.zip › cancers-1643078-supplementary.pdf]

Table S1. Search strategies for use in the databases.

Databases	***Search Strategy***
Medline/PubMed	*Search: (Curcumin OR CURCUMA longa OR Turmeric OR Natural yellow 3 OR Turmeric yellow OR Indian saffron OR Kacha haldi OR curcumin nanoparticles) AND (breast cancer OR Breast Neoplasms OR Triple Negative Breast Neoplasms OR Breast Tumor OR Inflammatory Breast Neoplasms OR Carcinoma, Ductal, Breast OR Carcinoma, Lobular OR HER-2 Positive Breast Cancer) AND (In vitro OR/AND mouse OR animal)*<br>***Total: 312***
EMBASE	*1 (Curcumin or CURCUMA longa or Turmeric OR Natural yellow 3 OR Turmeric yellow OR Indian saffron OR Kacha haldi OR curcumin nanoparticles).mp. [mp=title, abstract, heading word, drug trade name, original title, device manufacturer, drug manufacturer, device trade name, keyword, floating subheading word, candidate term word] 33231*<br>*2 (breast cancer or Breast Neoplasms or Triple Negative Breast Neoplasms or Breast Tumor or Inflammatory Breast Neoplasms or Carcinoma, Ductal, Breast or Carcinoma, Lobular or HER-2 Positive Breast Cancer).mp. [mp=title, abstract, heading word, drug trade name, original title, device manufacturer, drug manufacturer, device trade name, keyword, floating subheading word, candidate term word] 599531*<br>*3 ((In vitro and mouse) or animal).mp. [mp=title, abstract, heading word, drug trade name, original title, device manufacturer, drug manufacturer, device trade name, keyword, floating subheading word, candidate term word] 6460800*<br>*4 (In vitro or mouse or animal).mp. [mp=title, abstract, heading word, drug trade name, original title, device manufacturer, drug manufacturer, device trade name, keyword, floating subheading word, candidate term word] 8088931*<br>*5 1 and 2 and 3 and 4 435*<br>***Total: 435***
Scopus	*TITLE-ABS-KEY ((curcumin OR curcuma AND longa OR turmeric OR Natural yellow 3 OR Turmeric yellow OR Indian saffron OR Kacha haldi) AND (breast AND cancer)) AND (LIMIT-TO (DOCTYPE, "article"))*<br>***Total: 249***
Web of Science	*((Curcumin*) AND (breast cancer) AND in vitro AND in vivo)* **Total: 291**
Scielo	*Curcumin OR CURCUMA longa OR Turmeric*<br>*AND*<br>*breast cancer*<br>*AND*<br>*In vitro OR/AND mouse OR animal*<br>***Total: 1***

Table S2. Articles excluded and reasons for exclusion.

Number	Title	Reason for Exclusion
1	Abdel-Hafez SM, Hathout RM, Sammour OA. Curcumin-loaded ultradeformable nanovesicles as a potential delivery system for breast cancer therapy. Colloids Surf B Biointerfaces. 2018 Jul 1;167:63-72. doi: 10.1016/j.colsurfb.2018.03.051.	1
2	Abstract 2870: Preventive efficacy of curcumin on ENU-induced carcinogenic transformation of ApcMin/+ mammary epithelial (MinMG) cells established from ApcMin/+ mouse. Cancer Res April 15 2010 (70) (8 Supplement) 2870; DOI: 10.1158/1538-7445.AM10-2870	2
3	Delfan M, Rasekh Nejad Z, Delphan M. Synergistic Effect of Endurance Training Combined with Curcumin on Intratumoral Expression of Interleukin-4 (Il4) and Stat-6 in Female Mice with Breast Cancer. Iranian Quarterly Journal of Breast Disease. 2020; 13(3):52-61.	2
4	Ferreira, Lívia Carvalho et al. Abstract A02: Effect of curcumin on the tumor growth and angiogenesis of breast cancer. 2015. Cancer Research 75(1 Supplement):A02-A02	2
5	Ferreira et al.: Evaluation of the expression of angiogenic factors in breast cancer after curcumin treatment. BMC Proceedings 2013 7(Suppl 2):P40.	2
6	Gallardo M, Calaf GM. Curcumin and epithelial-mesenchymal transition in breast cancer cells transformed by low doses of radiation and estrogen. Int J Oncol. 2016 Jun;48(6):2534-42. doi: 10.3892/ijo.2016.3477.	3
7	Grill, Alex, and Jayanth Panyam. Abstract 1856: Sustained release curcumin microparticles delay mammary tumorigenesis in BALB-neuT transgenic mice. Cancer Research 2011: 1856-1856.	2
8	Huang L, Li A, Liao G, Yang F, Yang J, Chen X, Jiang X. Curcumol triggers apoptosis of p53 mutant triple-negative human breast cancer MDA-MB 231 cells via activation of p73 and PUMA. Oncol Lett. 2017 Jul;14(1):1080-1088. doi: 10.3892/ol.2017.6273.	3
9	Kusuhara H, Furuie H, Inano A, Sunagawa A, Yamada S, Wu C, Fukizawa S, Morimoto N, Ieiri I, Morishita M, Sumita K, Mayahara H, Fujita T, Maeda K, Sugiyama Y. Pharmacokinetic interaction study of sulphasalazine in healthy subjects and the impact of curcumin as an in vivo inhibitor of BCRP. Br J Pharmacol. 2012 Jul;166(6):1793-803. doi: 10.1111/j.1476-5381.2012.01887.x.	1
10	Liu, L., Fu, Y., Zheng, Y., Ma, M., & Wang, C. (2020). Curcumin inhibits proteasome activity in triple-negative breast cancer cells through regulating p300/miR-142–3p/PSMB5 axis. Phytomedicine, 78, 153312.	1
11	LIU, Zhongfa et al. Abstract 581: Complementary reactivation of tumor suppressor genes in breast cancer cells by curcumin and curcumin O-glucuronide. 2012.	2
12	Beatrice E. Bachmeier, Isabelle V. Mohrenz, Valentina Mirisola, Erwin Schleicher, Francesco Romeo, Clara Höhneke, Marianne Jochum, Andreas G. Nerlich, Ulrich Pfeffer, Curcumina regula negativamente as citocinas inflamatórias CXCL1 e -2 em células de câncer de mama via NFκB, Carcinogênese , Volume 29, Edição 4, abril de 2008, Páginas 779-789, https://doi.org/10.1093/carcin/bgm248	1

13	RAMACHANDRAN, Cheppail et al. Curcumin inhibits telomerase activity through human telomerase reverse transcritpase in MCF-7 breast cancer cell line. Cancer letters, v. 184, n. 1, p. 1-6, 2002.	1
14	SHEIKH, Eram et al. Bio-based synthesised and characterized monodispersed Curcuma longa silver nanoparticles induces targeted anticancer activity in breast cancer cells. Pharmacognosy Magazine, v. 14, n. 57, p. 340, 2018.	3
15	Aggarwal BB, Shishodia S, Takada Y, Banerjee S, Newman RA, Bueso-Ramos CE, Price JE. Curcumin suppresses the paclitaxel-induced nuclear factor-kappaB pathway in breast cancer cells and inhibits lung metastasis of human breast cancer in nude mice. Clin Cancer Res. 2005 Oct 15;11(20):7490-8. doi: 10.1158/1078-0432.CCR-05-1192.	1
16	de Sá, I. S., Peron, A. P., Leimann, F. V., Bressan, G. N., Krum, B. N., Fachinetto, R., ... & Ineu, R. P. (2019). In vitro and in vivo evaluation of enzymatic and antioxidant activity, cytotoxicity and genotoxicity of curcumin-loaded solid dispersions. Food and Chemical Toxicology, 125, 29-37.	1
17	Taurin S, Nehoff H, Diong J, Larsen L, Rosengren RJ, Greish K. Curcumin-derivative nanomicelles for the treatment of triple negative breast cancer. J Drug Target. 2013 Aug;21(7):675-83. doi: 10.3109/1061186X.2013.796955.	3
18	Wang Y, Yu J, Cui R, Lin J, Ding X. Curcumin in Treating Breast Cancer. J Lab Autom. 2016 Dec;21(6):723-731. doi: 10.1177/2211068216655524.	4
19	Van Long, N., Ha, B. T. T., Tuan, A. V., Van Luong, H., Linh, N. T., Duc, T. C., ... & Van Men, C. (2019). Phytosomal Nanoparticles Preparation of Curcuminoids to Enhance Cellular Uptake of Curcuminoids on Breast Cancer Cell Line MCF-7. Pharmacognosy Journal, 11(5).	3
20	Guo Y, Peng AB, Liao AJ, Shi W, Hu GS, Zhang LM. [Effect of zedoary turmeric oil on proliferation, apoptosis and extracellular matrix secretion of hepatic stellate cells]. Zhonghua Gan Zang Bing Za Zhi. 2008 Apr;16(4):302-3. Chinese. PMID: 18423156.	3
21	Zhang, Wenda, et al. "Discovery of monocarbonyl curcumin-BTP hybrids as STAT3 inhibitors for drug-sensitive and drug-resistant breast cancer therapy." *Scientific reports* 7.1 (2017): 1-17.	3
22	Carroll CE, Benakanakere I, Besch-Williford C, Ellersieck MR, Hyder SM. Curcumin delays development of medroxyprogesterone acetate-accelerated 7,12-dimethylbenz[a]anthracene-induced mammary tumors. Menopause. 2010 Jan-Feb;17(1):178-84. doi: 10.1097/gme.0b013e3181afcce5.	1
23	Chang CC, Fu CF, Yang WT, Chen TY, Hsu YC. The cellular uptake and cytotoxic effect of curcuminoids on breast cancer cells. Taiwan J Obstet Gynecol. 2012 Sep;51(3):368-74. doi: 10.1016/j.tjog.2012.07.009.	1
24	Deshpande, Shailesh S., Arvind D. Ingle, and Girish B. Maru. "Chemopreventive efficacy of curcumin-free aqueous turmeric extract in 7, 12-dimethylbenz [a] anthracene-induced rat mammary tumorigenesis." Cancer letters 123.1 (1998): 35-40.	5
25	Dong S, Alahari SK. Combination treatment of bicalutamide and curcumin has a strong therapeutic effect on androgen receptor-positive triple-negative breast cancers. Anticancer Drugs. 2020 Apr;31(4):359-367. doi: 10.1097/CAD.0000000000000880.	5

26	Calaf GM, Echiburú-Chau C, Wen G, Balajee AS and Roy D: Effect of curcumin on irradiated and estrogen-transformed human breast cell lines. Int J Oncol 40: 436-442, 2012	1
27	Fathy Abd-Ellatef GE, Gazzano E, Chirio D, Hamed AR, Belisario DC, Zuddas C, Peira E, Rolando B, Kopecka J, Assem Said Marie M, Sapino S, Ramadan Fahmy S, Gallarate M, Abdel-Hamid AZ, Riganti C. Curcumin-Loaded Solid Lipid Nanoparticles Bypass P-Glycoprotein Mediated Doxorubicin Resistance in Triple Negative Breast Cancer Cells. Pharmaceutics. 2020 Jan 24;12(2):96. doi: 10.3390/pharmaceutics12020096.	1
28	GHOSH, Shatadal et al. Targeted delivery of curcumin in breast cancer cells via hyaluronic acid modified mesoporous silica nanoparticle to enhance anticancer efficiency. Colloids and Surfaces B: Biointerfaces, v. 197, p. 111404, 2021.	5
29	Ibrahim A, El-Meligy A, Lungu G, Fetaih H, Dessouki A, Stoica G, Barhoumi R. Curcumin induces apoptosis in a murine mammary gland adenocarcinoma cell line through the mitochondrial pathway. Eur J Pharmacol. 2011 Oct 1;668(1-2):127-32. doi: 10.1016/j.ejphar.2011.06.048.	1
30	Khan MN, Haggag YA, Lane ME, McCarron PA, Tambuwala MM. Polymeric Nano-Encapsulation of Curcumin Enhances its Anti-Cancer Activity in Breast (MDA-MB231) and Lung (A549) Cancer Cells Through Reduction in Expression of HIF-1α and Nuclear p65 (Rel A). Curr Drug Deliv. 2018 Feb 14;15(2):286-295. doi: 10.2174/1567201814666171019104002.	1,5
31	Kim HI, Huang H, Cheepala S, Huang S, Chung J. Curcumin inhibition of integrin (alpha6beta4)-dependent breast cancer cell motility and invasion. Cancer Prev Res (Phila). 2008 Oct;1(5):385-91. doi: 10.1158/1940-6207.CAPR-08-0087.	1
32	Liu Y, Zhou J, Hu Y, Wang J, Yuan C. Curcumin inhibits growth of human breast cancer cells through demethylation of DLC1 promoter. Mol Cell Biochem. 2017 Jan;425(1-2):47-58. doi: 10.1007/s11010-016-2861-4.	1
33	Onoda M, Inano H. Effect of curcumin on the production of nitric oxide by cultured rat mammary gland. Nitric Oxide. 2000 Oct;4(5):505-15. doi: 10.1006/niox.2000.0305.	6
34	Bianchi G, Ravera S, Traverso C, Amaro A, Piaggio F, Emionite L, Bachetti T, Pfeffer U, Raffaghello L. Curcumin induces a fatal energetic impairment in tumor cells in vitro and in vivo by inhibiting ATP-synthase activity. Carcinogenesis. 2018 Sep 21;39(9):1141-1150. doi: 10.1093/carcin/bgy076.	1
35	Quispe-Soto ET and Quispe-Soto ET: Effect of curcumin and paclitaxel on breast carcinogenesis. Int J Oncol 49: 2569-2577, 2016	1
36	Thulasidasan AKT, Retnakumari AP, Shankar M, et al. Folic acid conjugation improves the bioavailability and chemosensitizing efficacy of curcumin-encapsulated PLGA-PEG nanoparticles towards paclitaxel chemotherapy. Oncotarget. 2017;8(64):107374-107389. Published 2017 Nov 10. doi:10.18632/oncotarget.22376	1
37	Rashidzadeh H, Rezaei SJT, Zamani S, Sarijloo E, Ramazani A. pH-sensitive curcumin conjugated micelles for tumor triggered drug delivery. J Biomater Sci Polym Ed. 2021 Feb;32(3):320-336. doi: 10.1080/09205063.2020.1833815.	1

38	Curcumin enhances the efficacy of chemotherapy by tailoring p65NFκB-p300 cross-talk in favor of p53-p300 in breast cancer. J Biol Chem. 2011 Dec 9;286(49):42232-42247. doi: 10.1074/jbc.M111.262295.	1
39	Shen Y, Han Z, Liu S, Jiao Y, Li Y, Yuan H. Curcumin Inhibits the Tumorigenesis of Breast Cancer by Blocking Tafazzin/Yes-Associated Protein Axis. Cancer Manag Res. 2020 Feb 27;12:1493-1502. doi: 10.2147/CMAR.S246691. Retraction in: Cancer Manag Res. 2020 Jul 14;12:5739. PMID: 32161501; PMCID: PMC7051254.	7
40	Somasundaram S, Edmund NA, Moore DT, Small GW, Shi YY, Orlowski RZ. Dietary curcumin inhibits chemotherapy-induced apoptosis in models of human breast cancer. Cancer Res. 2002 Jul 1;62(13):3868-75. PMID: 12097302.	1
41	Zhou QM, Su SB, Zhang H, Lu YY. [Regulation of protein kinases on signal pathway in breast cancer cell MCF-7 by curcumin]. Zhong Yao Cai. 2009 May;32(5):728-32.	1
42	Zhou, X, Jiao, D, Dou, M, et al. Curcumin inhibits the growth of triple-negative breast cancer cells by silencing EZH2 and restoring DLC1 expression. J Cell Mol Med. 2020; 24: 10648– 10662. https://doi.org/10.1111/jcmm.15683	1
43	Purushothaman, Bindhya K., P. Uma Maheswari, and KM Meera Sheriffa Begum. "Magnetic assisted curcumin drug delivery using folate receptor targeted hybrid casein-calcium ferrite nanocarrier." Journal of Drug Delivery Science and Technology 52 (2019): 509-520.	1
44	Singletary K, MacDonald C, Wallig M, Fisher C. Inhibition of 7,12-dimethylbenz[a]anthracene (DMBA)-induced mammary tumorigenesis and DMBA-DNA adduct formation by curcumin. Cancer Lett. 1996 Jun 5;103(2):137-41. doi: 10.1016/0304-3835(96)04224-3.	4

Reason for exclusion:

1Outcome different from those proposed in the study;

2 Congress abstract available only;

3 Use of other drug or component;

4 Type of study;

5 Association of curcumin to other component;

6 Type of tumour;

7 Retracted article.

Table S3. Quality of evidence in the preclinical studies.

Authors	GRADE								
	Study Design	Study Limitations	Inconsistency	Indirectness	Imprecision	Risk of Bias/Publication Biases	Magnitude of Moderate/Significant Effect	Effect of Dose	Overall Quality
Abd-Ellatef et al., 2020 [34]	Preclinical studies, animal model	√	√	√	√	√	√	√	++++
Alizadeh et al., 2015 [25]	Preclinical studies, animal model	√	√	√	√	√	√	√	++++
Abbaspour & Afshar, 2018 [23]	*In vitro* assay	X: Lack of results for some of the outcomes	√	√	√	√	√	√	+++
Abuelba et al., 2015 [24]	*In vitro* assay	√	√	√	√	√	√	√	++++
Bansal et al., 2014 [26]	Preclinical studies, animal model	√	√	√	√	√	√	√	++++
Bimonte et al., 2015 [7]	Preclinical study, both *in vitro* and animal model	X: Lack of results for some of the outcomes	√	√	√	√	√	√	+++
Calaf et al., 2018 [27]	*In vitro* assay	X: Lack of results for some of the outcomes	√	√	√	√	√	√	+++
Chen et al., 2017 [28]	Preclinical studies, animal model	√	√	√	√	√	√	√	++++
Chiu & Su, 2009 [29]	*In vitro* assay	X: Lack of results for some of the outcomes	√	√	√	√	√	√	+++
Choudhuri et al., 2002 [30]	*In vitro* assay	X: Lack of results for some of the outcomes	√	√	√	√	√	√	+++
Coker-Gurkan et al., 2019 [31]	*In vitro* assay	X: Lack of results for some of the outcomes	√	√	√	√	√	√	+++
Coker-Gurkan et al., 2018 [32]	*In vitro* assay	X: Lack of results for some of the outcomes	√	√	√	√	√	√	+++
Fan et al., 2016 [33]	*In vitro* assay	X: Lack of results for some of the outcomes	√	√	√	√	√	√	+++
Ghosh et al., 2021 [35]	Preclinical study, both *in vitro* and animal model	X: Lack of results for some of the outcomes	√	√	√	√	√	√	+++
Greish et al., 2018 [36]	Preclinical studies, animal model	√	√	√	√	√	√	√	++++
Grill et al., 2018 [37]	Preclinical studies, animal model	√	√	√	√	√	√	√	++++
Hashemzehi et al., 2018 [38]	Preclinical study, both *in vitro* and animal model	X: Lack of results for some of the outcomes	√	√	√	√	√	√	+++
He et al., 2019 [39]	Preclinical study, both *in vitro* and animal model	X: Lack of results for some of the outcomes	√	√	√	√	√	√	+++
Hu et al., 2018 [40]	*In vitro* assay	X: Lack of results for some of the outcomes	√	√	√	√	√	√	+++
Huang et al., 2020 [42]	Preclinical studies, animal model	√	√	√	√	√	√	√	++++
Hua et al., 2010 [41]	*In vitro* assay	X: Lack of results for some of the outcomes	√	√	√	√	√	√	+++
Ji et al., 2020 [43]	Preclinical study, both *in vitro* and using animal model	X: Lack of results for some of the outcomes	√	√	√	√	√	√	+++
Jiang et al., 2013 [44]	*In vitro* assay	X: Lack of results for some of the outcomes	√	√	√	√	√	√	+++

Jin et al., 2017 [45]	Preclinical study, both *in vitro* and animal model	X: Lack of results for some of the outcomes	√	√	√	√	√	√	+++
Jung et al., 2018 [46]	Preclinical study, both *in vitro* and animal model	X: Lack of results for some of the outcomes	√	√	√	√	√	√	+++
Kim et al., 2012 [47]	*In vitro* assay	X: Lack of results for some of the outcomes	√	√	√	√	√	√	+++
Kumari et al., 2017 [48]	*In vitro* assay	X: Lack of results for some of the outcomes	√	√	√	√	√	√	+++
Kumari et al., 2020 [49]	Preclinical study, both *in vitro* and animal model	X: Lack of results for some of the outcomes	√	√	√	√	√	√	+++
Kumari et al., 2016 [50]	*In vitro* assay	X: Lack of results for some of the outcomes	√	√	√	√	√	√	+++
Laha et al., 2018 [51]	Preclinical study, both *in vitro* and animal model	X: Lack of results for some of the outcomes	√	√	√	√	√	√	+++
Lai et al., 2012 [52]	Preclinical study, both *in vitro* and using animal model	X: Lack of results for some of the outcomes	√	√	√	√	√	√	+++
Li et al., 2018 [3]	Preclinical study, both *in vitro* and animal model	X: Lack of results for some of the outcomes	√	√	√	√	√	√	+++
Liu et al., 2013 [54]	Preclinical study, both *in vitro* and animal model	X: Lack of results for some of the outcomes	√	√	√	√	√	√	+++
Liu et al., 2009 [55]	*In vitro* assay	X: Lack of results for some of the outcomes	√	√	√	√	√	√	+++
Lin et al., 2016 [53]	Preclinical studies, animal model	√	√	√	√	√	√	√	++++
Lv et al., 2014 [57]	Preclinical studies, both *in vitro* and animal model	X: Lack of results for some of the outcomes	√	√	√	√	√	√	+++
Lv et al., 2015 [56]	Preclinical studies, animal model	√	√	√	√	√	√	√	++++
Mahalunkar et al., 2019 [58]	Preclinical studies, animal model	√	√	√	√	√	√	√	++++
Masuelli et al., 2013 [59]	Preclinical study, both *in vitro* and animal model	X: Lack of results for some of the outcomes	√	√	√	√	√	√	+++
Mehta et al., 1997 [60]	*In vitro* assay	X: Lack of results for some of the outcomes	√	√	√	√	√	√	+++
Montazeri et al., 2017 [61]	*In vitro* assay	X: Lack of results for some of the outcomes	√	√	√	√	√	√	+++
Mukhopadhyay et al., 2020 [62]	Preclinical study, both *in vitro* and animal model	X: Lack of results for some of the outcomes	√	√	√	√	√	√	+++
Mukerjee et al., 2016 [63]	Preclinical studies, animal model	√	√	√	√	√	√	√	++++
Pal et al., 2019 [64]	Preclinical studies, animal model	√	√	√	√	√	√	√	++++
Sahne et al., 2019 [65]	Preclinical studies, animal model	√	√	√	√	√	√	√	++++
Sarighieh et al., 2020 [66]	*In vitro* assay	X: Lack of results for some of the outcomes	√	√	√	√	√	√	+++
Shiri et al., 2015 [67]	Preclinical studies, animal model	√	√	√	√	√	√	√	++++
Shukla et al., 2017 [68]	Preclinical studies, animal model	√	√	√	√	√	√	√	++++

Sun Shih-Han et al., 2012 [69]	*In vitro* assay	X: Lack of results for some of the outcomes	√	√	√	√	√	√	+++
Sun Xiao-Dong et al., 2012 [70]	*In vitro* assay	X: Lack of results for some of the outcomes	√	√	√	√	√	√	+++
Vakilinezhad et al., 2019 [71]	Preclinical studies, animal model	√	√	√	√	√	√	√	++++
Wang et al., 2018 [73]	Preclinical studies, animal model	√	√	√	√	√	√	√	++++
Wang Xet al., 2017 [74]	*In vitro* assay	X: Lack of results for some of the outcomes	√	√	√	√	√	√	+++
Yang et al., 2017 a [74]	Preclinical study, both *in vitro* and animal model	X: Lack of results for some of the outcomes	√	√	√	√	√	√	+++
Yang et al., 2017 b [75]	Preclinical studies, animal model	√	√	√	√	√	√	√	++++
Younesian et al., 2017 [76]	*In vitro* assay	X: Lack of results for some of the outcomes	√	√	√	√	√	√	+++
Yu et al., 2021 [77]	Preclinical study, both *in vitro* and animal model	X: Lack of results for some of the outcomes	√	√	√	√	√	√	+++
Yu et al., 2014 [78]	Preclinical studies, animal model	√	√	√	√	√	√	√	++++
Yuan et al., 2018 [79]	Preclinical studies, animal model	√	√	√	√	√	√	√	++++
Zong et al., 2012 [80]	*In vitro* assay	X: Lack of results for some of the outcomes	√	√	√	√	√	√	+++

Evaluation: √, no sensible limitations; X, Sensible/severe limitations (or absent for magnitude of moderate/great effect, magnitude of dose effect, and effect of dose); Unclear, item non-classifiable based on available information.

Overall quality of evidence: +, very low; ++, low; +++, moderate; ++++, high.

Consideration for *in vitro* studies:

Consideration for animal model studies:

(1) Risk of bias: the SYRCLE tool could be used to assess the risk of bias in animal studies. (2) Indirectness: indirect assessment of animal studies using the PICO approach. (3) Inconsistency: similarity of point estimation, extension of superposition of confidence intervals (ICs), and statistical heterogeneity are adequate for assessing evidence inconsistency in animal studies. (4) Imprecision: the ideal magnitude of information and the 95% ICs are adequate for the systematic reviews of both animal study and clinical assays. (5) Publication bias: the publication bias considered comprehensively by qualitative and quantitative methods.
